# Integrating Critical Racial Literacy to Advance Health Equity Among African, Caribbean, and Black Populations in High-Income Countries: Protocol for a Scoping Review

**DOI:** 10.2196/79361

**Published:** 2026-06-11

**Authors:** Josephine Bassey Etowa, Arone W Fantaye, Aida Bahramian, Akalewold T Gebremeskel, Ubabuko Unachukwu, Amos Buh

**Affiliations:** 1 School of Nursing Faculty of Health Sciences University of Ottawa Ottawa, ON Canada; 2 School of International Development and Global Studies University of Ottawa Ottawa, ON Canada; 3 Canadians of African Descent Health Organization (CADHO) Ottawa, ON Canada; 4 Collaborative Critical Research for Equity and Transformation in Health Lab Faculty of Health Sciences University of Ottawa Ottawa, ON Canada; 5 Interdisciplinary School of Health Sciences Faculty of Health Sciences University of Ottawa Ottawa, ON Canada; 6 School of Human Kinetics Faculty of Health Sciences University of Ottawa Ottawa, ON Canada; 7 Canadian Red Cross Society Ottawa, ON Canada; 8 Telfer School of Management University of Ottawa Ottawa, ON Canada; 9 Faculty of Health Sciences University of Buea Buea Cameroon

**Keywords:** critical race literacy, health equity, African, Caribbean, and Black populations, anti-Black racism, structural racism, scoping review

## Abstract

**Background:**

African, Caribbean, and Black (ACB) populations in high-income countries (HICs) continue to experience long-standing health inequities rooted in structural and anti-Black racism embedded in health systems, policies, and institutional practices. From an ecosocial perspective, these inequities reflect the embodiment of intersecting forms of oppression structured through racialized, gendered, and socioeconomic relations. Critical racial literacy (CRL) has emerged as a promising framework for recognizing and addressing structural racism in ways that foster critical reflection and support justice-oriented action in health contexts. However, evidence on how CRL is conceptualized and operationalized in health research, policy, and practice concerning ACB communities remains fragmented and limited.

**Objective:**

This scoping review aims to map how CRL is conceptualized and operationalized in HICs and examine its potential to advance health equity for ACB populations.

**Methods:**

This scoping review will be conducted in accordance with the Joanna Briggs Institute guidance. Comprehensive searches will be conducted in MEDLINE (Ovid), Embase, CINAHL, PsycInfo, ERIC, Scopus, and ProQuest Dissertations and Theses Global from inception to March 31, 2026. Peer-reviewed articles and theses or dissertations will be included, with no restrictions on study design or publication type. At least 2 independent reviewers will conduct screening, charting, and analysis of the data. A 3-phase thematic mapping process guided by critical race theory, intersectionality, and ecosocial theory will be used to analyze and interpret the findings.

**Results:**

Searching, screening, data charting, and analysis will be undertaken between April 2026 and July 2026. Manuscript preparation will be completed by July 31, 2026, and dissemination will occur between August 2026 and October 2026. The findings will identify key CRL components, applications, strategies, barriers, and equity pathways across clinical, policy, and community contexts.

**Conclusions:**

This scoping review will provide a comprehensive overview of how CRL is conceptualized and applied in health contexts involving ACB populations in HICs. By clarifying current conceptualizations, applications, and gaps in the literature, the review will identify priorities for future theoretical, methodological, and practice-based development. In doing so, the findings will inform more critically grounded, praxis-oriented, and structurally focused antiracism efforts across health systems. Results will be disseminated through open access publications, conference presentations, and stakeholder engagement activities to advance evidence-informed health equity action.

**Trial Registration:**

PROSPERO CRD42024623132; https://www.crd.york.ac.uk/PROSPERO/view/CRD42024623132

**International Registered Report Identifier (IRRID):**

PRR1-10.2196/79361

## Introduction

Racial inequities in health and health care remain a persistent and consequential structural challenge across high-income countries (HICs) [1-4]. African, Caribbean, and Black (ACB) populations continue to experience disproportionate burdens of illness and premature mortality alongside persistent barriers to timely, appropriate, and high-quality care [5-8]. These inequities are neither incidental nor adequately explained by individual behavior, culture, or access alone. Rather, they are produced and sustained through historical processes of racialization and structural racism that shape exposure to discrimination, adverse social and material conditions and health care experiences, and access to the resources and opportunities necessary for health [1,2,9-14]. In this sense, such inequities reflect the embodiment of historically and institutionally structured forms of racialized disadvantage across the life course [2,12,15]. These dynamics became particularly visible during the COVID-19 pandemic, when ACB communities in Canada and elsewhere experienced disproportionate risks and compounded structural barriers to prevention, diagnosis, and care [7,16-19].

Despite growing recognition of these inequities, dominant institutional responses have often remained limited in scope and effect. Equity, diversity, and inclusion initiatives, as well as conventional health literacy approaches, have frequently emphasized representation, organizational messaging, awareness, or individual knowledge acquisition without sufficiently confronting the structural relations of power through which racial inequities are reproduced [20-24]. Although such responses may have procedural value, they are often substantively inadequate for addressing anti-Black racism when they are not linked to institutional accountability, material change, and structural transformation [25-27]. Health literacy interventions have similarly made important contributions to communication and patient education, particularly in community-based settings, but they have generally not been designed to interrogate racism as a systemic determinant of health or challenge the institutional and social conditions through which it is reproduced [28-30]. Together, these limitations underscore the need for a framework that is explicitly antiracist, theoretically grounded, and capable not only of identifying racism within health care institutions and systems but also of examining how it is translated into inequitable health experiences and outcomes for ACB populations.

Critical race theory (CRT) provides an important foundation for such work. CRT conceptualizes race not as a biological fact but as a social and political construction produced through law, institutions, and historical relations of power [31,32]. From this perspective, racism is ordinary, systemic, and embedded within the social order rather than exceptional or reducible to individual prejudice [9,11,32]. CRT also challenges dominant assumptions of neutrality, color blindness, and objectivity that can obscure racial power while centering social justice, counterstorytelling, reflexive critique, and structural transformation as essential to both analysis and action [32-35]. In health research and practice, CRT is especially valuable because it enables more direct analysis of how inequities are structured, justified, and reproduced across policy, institutions, and everyday practice [27,33].

Building on these foundations, critical racial literacy (CRL) has emerged as a promising conceptual and analytic framework for identifying, interpreting, and challenging the operation of racism across discourse, policy, professional practice, and organizational life [36-40]. Unlike broader formulations of racial literacy, CRL foregrounds critical consciousness, historical and sociopolitical situated analysis, reflexive engagement with positionality, structural interpretation, and sustained antiracist praxis [37-39]. Its relevance in health is especially significant because health care institutions often reproduce racism in ways that are diffuse, normalized, and difficult to name within dominant organizational frameworks of professionalism, neutrality, and equity [2,12,41]. In this context, CRL is important not simply because it helps identify racial inequities but also because it offers interpretive tools for examining how racism is obscured, legitimized, and reproduced through institutional discourse, policy logics, professional norms, and routine practices [20,21,25-27,33,42,43]. By rendering visible forms of racial silence, race evasiveness, normalization, and institutional denial, CRL makes it possible to interrogate how anti-Black racism may persist even within systems that formally espouse equity [23,36,39]. In HICs, where health systems often publicly commit to equity while continuing to produce racialized exclusions and uneven health outcomes, CRL may be especially useful for interrogating the gap between institutional commitments and lived realities.

CRL is also particularly important in health because it links the analysis of racism to its material and embodied consequences. Rather than treating inequities in health care access, quality, and outcomes as isolated disparities, CRL situates them within the broader structural conditions through which racism shapes exposure to harm; constrains opportunities for health; and organizes differential relationships to care, recognition, and institutional accountability [24,32,33,36,37,39,40]. In doing so, CRL has the potential to extend analysis beyond the descriptive documentation of inequities toward a more critical examination of how racism is conceptualized, operationalized, and challenged across health research, policy, and practice [36,39,40,43]. Therefore, for the purposes of this review, CRL is approached as a potentially valuable framework for examining conceptual gaps, institutional tensions, and possibilities for structural transformation in relation to ACB populations in ways that may not be fully captured by broader antiracism or general health equity approaches.

The relevance of CRL is especially pronounced in relation to ACB populations, whose health experiences are shaped not only by racism in general terms but also by anti-Black racism as a historically specific and structurally entrenched formation linked to slavery, colonialism, and racial capitalism [9,25,31,44]. Within health systems, anti-Black racism has been associated with mistrust, delayed care, discriminatory encounters, and forms of institutional neglect that undermine equitable access and outcomes [42,45-51]. Situated within a broader theoretical constellation that includes CRT, intersectionality, and ecosocial theory, CRL offers a useful lens for understanding how structurally produced racism may be differentially experienced, embodied, and reproduced across social positions and institutional contexts [2,32,36,37,42,52].

At the same time, CRL in health remains an emergent field of scholarship. Although a small but important body of work has begun to articulate its relevance for anti-Black racism, health equity, and institutional accountability [20,33,36,39,43], the literature remains limited in scope and concentrated within a relatively narrow set of contributions. There is still no clear synthesis of how CRL has been defined, what its core dimensions are in health contexts, or how it has been implemented and operationalized in relation to ACB populations in HICs [36,39,40,43]. This lack of synthesis makes it difficult to assess the conceptual coherence, practical applications, and future directions of this emerging body of work. Moreover, although a growing body of scholarship has documented anti-Black racism in health care and evaluated broader antiracism and equity-oriented interventions [20,21], relatively few studies have conceptualized or applied CRL as a distinct, praxis-oriented framework for institutional transformation, accountability, or community-engaged health equity practice [37,39,40].

Therefore, a scoping review is warranted because it is particularly well suited to emerging and conceptually heterogeneous fields in which definitions, dimensions, and applications remain underdeveloped [53,54]. Accordingly, this review asks the following: how is CRL conceptualized and operationalized in HICs to advance health equity for ACB populations? Its objectives are to map (1) how CRL is defined and conceptualized in health contexts; (2) the principal dimensions, components, and practices associated with CRL in the literature; and (3) the ways in which CRL has been operationalized to address structural racism and advance health equity for ACB populations in HICs. By consolidating this emerging body of scholarship, the review will strengthen conceptual clarity, identify key gaps, and inform the development of more rigorous and structurally grounded antiracist approaches to health equity.

## Methods

### Study Design

This scoping review follows the Joanna Briggs Institute methodology for scoping reviews [53]. Consistent with this approach, the review will involve the identification of relevant evidence, screening and selection of sources, data charting, and synthesis and presentation of results. Review processes will be iterative and team based, with regular consultation among the research team to support methodological consistency, reflexivity, and interpretive rigor. This protocol is reported in accordance with the PRISMA-ScR (Preferred Reporting Items for Systematic Reviews and Meta-Analyses extension for Scoping Reviews) alongside guidance for the development of scoping review protocols by Peters et al [55]. The review has been registered with PROSPERO (registration number: CRD42024623132).

### Theoretical Framework

This scoping review is grounded in CRT as its foundational lens and informed by CRL as its primary analytical framework. Within this review, CRT provides the structural basis for understanding race as socially constructed and racism as systemic, historically produced, and embedded within institutions and social relations rather than reducible to individual prejudice [9,11,32,56,57]. It also draws attention to how dominant claims of neutrality, objectivity, and color blindness can obscure racial power and institutional accountability [32-35]. Accordingly, CRT anchors the review’s focus on structural racism, the experiences of ACB populations, and the institutional production of health inequities.

Building on this foundation, CRL serves as the core interpretive framework. It is concerned with how racism is recognized, interpreted, and challenged across discourse, policy, professional practice, and organizational life [36,37,40,58]. In contrast to broader formulations of racial literacy, CRL emphasizes critical consciousness, historical and sociopolitical analysis, reflexive attention to positionality, structural analysis, and sustained antiracist action [36,37,40,59]. In health contexts, CRL is particularly valuable because it illuminates forms of racial silence, race evasiveness, normalization, and institutional denial while also providing a framework for analyzing how racism structures inequitable health care experiences, constrains opportunities for health, and contributes to disparities in health outcomes among ACB populations [22,29,30,39,47,52,60]. In this review, the specific contribution of CRL lies in its capacity to move the analysis beyond documenting inequities toward examining how racism is conceptualized, normalized, and challenged across health research, policy, and practice.

To deepen interpretation of the findings, the review also draws on intersectionality and ecosocial theory as complementary analytic lenses. Intersectionality highlights how racism intersects with gender, socioeconomic position, migration status, colorism, and other relations of power to produce heterogeneous experiences within and across ACB communities [31,42,56,61]. Ecosocial theory further informs the analysis by explaining how structurally produced racism becomes embodied through historically and institutionally organized pathways of exposure, exclusion, constrained opportunity, and differential accountability across the life course [2,62,63]. Together, these lenses broaden the analysis to the heterogeneity of racialized experience, the operation of institutional processes, and the embodiment of inequities.

Taken together, CRT, CRL, intersectionality, and ecosocial theory guide the review’s synthesis in complementary ways [2,32,33,36,40,52]. CRT anchors the structural analysis of racism; CRL guides the mapping and interpretation of how racism is conceptualized and operationalized in the literature; and intersectionality and ecosocial theory deepen analysis of differential experience, institutional power, and embodied inequities. Collectively, these frameworks support a synthesis attentive not only to descriptive patterns in the literature but also to the relations of power and structural conditions through which health inequities are produced and sustained.

### Eligibility Criteria

This review will apply eligibility criteria structured according to the population, interest, context, and source type (PICoS) framework to identify literature relevant to how CRL is defined, conceptualized, and operationalized in health-related contexts. The criteria are designed to map the principal dimensions, practices, and applications of CRL, as well as the ways in which it has been mobilized to address structural racism and advance health equity for ACB populations in HICs (Table 1).

**Table 1:** Eligibility criteria for the review.

PICoS^a^ element	Inclusion criteria	Exclusion criteria
Population	Articles focused on or explicitly relevant to ACB^b^ populations or organizations. In this review, ACB refers inclusively “to individuals and communities who identify as Black and trace their heritage to sub-Saharan Africa, the Caribbean, or the African diaspora” [64]. Eligible populations and organizations may include ACB service users, communities, health care providers, and trainees or ACB-led organizations. Empirical and nonempirical sources involving multiple racialized populations will be included only where findings relevant to ACB populations are reported separately; analyzed as a distinct subgroup; or otherwise clearly identifiable through disaggregated data, quotations, case examples, or discussion specific to ACB populations. For the purposes of this review, this includes substantive ACB-specific description, analysis, theorization, interpretation, or implications relevant to CRL^c^ and the advancement of health equity.	Articles with no relevance to ACB populations, communities, or organizations. Studies or conceptual papers involving multiple racialized groups will be excluded where content relevant to ACB populations is not reported separately or cannot be clearly distinguished from content related to other groups.
Interest	Articles that define, conceptualize, implement, or operationalize CRL in health-related contexts. This includes literature addressing the dimensions, components, or practices associated with CRL, such as critical consciousness, historical and sociopolitical analysis, reflexive attention and positionality, structural analysis, interrogation of power relations, and antiracist action. Articles examining barriers, facilitators, strategies, or approaches relevant to the integration or operationalization of CRL in policy, programs, practice, education, or organizational change will also be included.	Articles with no conceptual, theoretical, or empirical relevance to racial literacy or CRL. Articles addressing racism, antiracism, or equity initiatives in general will be excluded unless they explicitly relate to CRL, racial literacy, or closely related conceptual elements central to CRL.
Context	Health-related contexts in HICs^d^ as defined by the World Bank’s current fiscal year income classification based on GNI^e^ per capita thresholds [64,65]. Where relevant, OECD^f^ member country status will be used to support identification of eligible contexts. They may include clinical care, public health, health promotion, health policy, health care organizations, organizational antiracism initiatives, and health professional education and training.	Studies conducted in low- or middle-income settings or in contexts not meaningfully related to health, health care, public health, or health systems.
Source type	Peer-reviewed empirical studies, review articles, conceptual or argument papers, commentaries, opinion pieces, and relevant gray literature (eg, graduate theses or dissertations, policy documents, organizational reports, practice frameworks, training resources, and implementation toolkits) that make a substantive contribution to understanding the conceptualization, dimensions, practices, or operationalization of CRL in relation to health equity for ACB populations.	Posters, conference abstracts, letters to the editor, study protocols, and sources lacking sufficient substantive detail for screening and data extraction.
Language	English and French	Sources for which the full text is available only in languages other than English or French
Publication date	No date restrictions	—^g^

^a^PICoS: population, interest, context, and source type.

^b^ACB: African, Caribbean, and Black.

^c^CRL: critical racial literacy.

^d^HIC: high-income country.

^e^GNI: gross national income.

^f^OECD: Organisation for Economic Co-operation and Development.

^g^Not applicable.

Following establishment of the eligibility criteria, a comprehensive search strategy will be developed to identify relevant published and unpublished literature across selected bibliographic databases and gray literature sources.

### Information Sources and Search Strategy

A comprehensive and sensitive multi-database search strategy will be developed and conducted by the review team in collaboration with an experienced health sciences librarian to identify relevant scholarly and gray literature. For scholarly literature, the following electronic databases will be searched: MEDLINE ALL via Ovid, Embase and Embase Classic via Ovid, CINAHL via EBSCOhost, APA PsycInfo via Ovid, Scopus, and ERIC. Ovid currently provides access to MEDLINE, Embase, Embase Classic, and APA PsycInfo on its platform [65].

A 3-step search strategy will be used. First, an initial limited search of MEDLINE ALL via Ovid will be undertaken to identify relevant articles and examine the keywords used in titles and abstracts, as well as the index terms assigned to relevant records. The preliminary MEDLINE search strategy will include a broad range of subject headings, such as “Racism” and “Health Equity,” along with keywords related to “Critical Racial Literacy,” “racial literacy,” “critical consciousness,” “anti-racism,” “structural racism,” “power,” “African, Caribbean, and Black populations,” and “health-related contexts.” Second, the MEDLINE search strategy will be refined and translated for use across the remaining bibliographic and gray literature sources. Third, all databases will be searched from inception to March 31, 2026. Moreover, the reference lists of all included sources will be screened to identify further relevant records not retrieved through database searching (see Multimedia Appendix 1 for the MEDLINE search strategy).

Because this review examines how CRL is conceptualized and operationalized across health research, policy, practice, and community contexts, targeted gray literature searches will also be conducted. These searches will be used to identify relevant policy documents, organizational reports, training resources, implementation frameworks, practice guidance, and toolkits. Gray literature sources will include government and public health websites, professional associations, health care organizations, Black-serving community organizations, and other relevant institutional websites in HICs. Gray literature searching will include ProQuest Dissertations and Theses Global, Google Scholar, the Canadian Agency for Drugs and Technologies in Health’s Grey Matters tool, and targeted website searching of relevant organizations, together with hand searching of identified documents and reference lists where appropriate. The Grey Matters tool remains an active resource for health-related gray literature searching, whereas OpenGrey was discontinued in December 2020 and preserved as an archive rather than maintained as a current database [66].

### Screening and Selection Process

Records identified through the searches will be imported into the Covidence software (Veritas Health Innovation) for deduplication and screening. Screening will be conducted in 2 stages: title and abstract screening followed by full-text review. At each stage, records will be assessed independently by 2 reviewers against the population, interest, context, and source type–based inclusion and exclusion criteria (Table 1). For studies involving multiple racialized populations, inclusion will require that findings relevant to ACB communities be reported separately, analyzed as a distinct subgroup, or otherwise clearly identifiable through disaggregated qualitative or quantitative data. Where relevance to ACB populations is uncertain at the title and abstract stage, the record will be retained for full-text review. During full-text screening, decisions regarding whether findings can be meaningfully distinguished for ACB populations will be guided by prespecified criteria and discussed among reviewers to support consistency.

Disagreements that arise at any point in the screening process will be resolved through discussion between the 2 reviewers and, where necessary, through consultation with the senior author (JBE) or adjudication by a third reviewer. The reasons for exclusion during the full-text stage will be documented, and the study selection process will be reported in a PRISMA-ScR flow diagram [67,68].

### Data Extraction and Items

A data extraction instrument will be developed by the review team in Microsoft Excel. To ensure alignment with the review question and objectives, data will be charted across seven primary domains: (1) bibliometric and source characteristics, including title, year, journal or source, and country of origin; (2) study or paper context, including publication type, population of focus, health-related setting, and methodological characteristics where applicable; (3) CRL definitions and conceptualizations; (4) the principal dimensions, components, and practices associated with CRL; (5) the ways in which CRL is implemented, integrated, or operationalized in health-related contexts; (6) reported barriers, facilitators, challenges, and strategies related to the operationalization of CRL in addressing structural racism and advancing health equity; and (7) implications for future research, policy, practice, and programmatic development. For studies involving multiple racialized populations, the extraction form will also capture how ACB populations were identified and analytically distinguished, including through separate subgroup analyses, disaggregated quantitative findings, qualitative quotations, case examples, or explicit ACB-specific discussion. Where extraction fields are not applicable to a given source type, these will be recorded as “not applicable.”

To calibrate the charting process, the review team will pilot the extraction form on a small sample of included sources and compare results through discussion to assess clarity, consistency, and alignment with the review objectives. The form will be refined iteratively as needed throughout the charting process. Data extraction will then be conducted by at least 2 reviewers, with uncertainties or discrepancies resolved through discussions and, if required, consultation with the senior author (JBE). Where relevant and feasible, the corresponding authors of included papers may be contacted to clarify ambiguous or missing information [53].

### Analysis and Reporting

Analysis and reporting will proceed in 2 stages. First, the included sources will be descriptively mapped to provide an overview of the evidence base. Data will be presented in summary tables derived from the data charting form with a brief narrative summary to describe the scope, distribution, and general article characteristics of the included literature.

Second, thematic mapping, adapted from the 3-phase approach described by Etowa et al [69], will be used to collate, interpret, and synthesize key patterns across the included sources. This approach is appropriate for a scoping review of an emerging and conceptually heterogeneous field because it supports the integration of diverse forms of evidence while maintaining descriptive breadth and analytic depth. In this review, thematic mapping will be used to examine how CRL is defined and conceptualized in health contexts; the principal dimensions, components, and practices associated with CRL; and the ways in which CRL has been operationalized to address structural racism and advance health equity for ACB populations in HICs.

Thematic mapping will proceed in 3 phases [69]. In phase 1 (individual study analysis), initial codes and descriptive themes will be developed from the extracted data. This phase will identify how CRL is framed across the literature, including variation in definitions, conceptual boundaries, dimensions, practices, and reported applications. Coding will be informed by the review’s theoretical framework, including CRT, CRL, intersectionality, and ecosocial theory. In phase 2 (within-group analysis), descriptive themes will be compared, interpreted, and refined into broader analytic themes through iterative examination of similarities and differences across the included sources. This phase will support a deeper analysis of how CRL is articulated in the literature, which dimensions and practices are emphasized or underdeveloped, and how operational applications are described in relation to health equity for ACB populations in HICs. In phase 3 (cross-group analysis), higher-order themes will be developed across the included evidence base to generate a synthesized account of CRL conceptualizations, dimensions, and operational applications. These themes will also support identification of conceptual, methodological, and practice-based gaps in the literature. Theme development will occur iteratively through comparison, discussion, and refinement within the research team to enhance consistency and interpretive rigor. The final findings will be presented in tables; a thematic narrative summary; and, where appropriate, a visual thematic map illustrating relationships across CRL conceptualizations, dimensions, and operational applications.

### Ethical Considerations

This protocol does not require ethics approval as it is based solely on the analysis of publicly available secondary documents. No primary data collection involving human participants will be conducted.

## Results

As of March 20, 2026, protocol development, integration of the theoretical framework, and registration with PROSPERO (CRD42024623132) had been completed. Following completion of the database searches, screening, eligibility assessment, data charting, and analysis are expected to take place between April 2026 and July 2026. It is anticipated that the completed review will provide a descriptive and thematic synthesis of the literature on CRL in health-related contexts involving ACB populations in HICs. Manuscript preparation will be completed by July 31, 2026, with submission of the final manuscript expected between August 2026 and October 2026.

## Discussion

### Anticipated Findings

This review will examine how CRL is defined, conceptualized, and operationalized across health-related contexts involving ACB populations in HICs. Given the emergent nature of this field, the review is likely to identify a limited and conceptually heterogeneous body of literature. The findings may reveal variation in how CRL is defined and framed across studies, as well as inconsistency in the dimensions, components, and practices associated with it. The review may also indicate that, although CRL is increasingly recognized as a promising framework for addressing structural racism in health, its application remains uneven and underdeveloped across policy, training, organizational, public health, and clinical settings.

The review is also expected to identify important gaps in the literature, including inconsistency in terminology, insufficient articulation of CRL dimensions in health contexts, and limited clarity regarding how CRL is translated into practice. In addition, the evidence base may be concentrated within a relatively small number of contributions, with few studies documenting applied or practice-based examples specifically with ACB populations. Studies that include broader racialized populations will be limited to sources in which findings related to ACB populations are clearly identifiable and can be meaningfully distinguished. By mapping these patterns, the review will help clarify the current state of the field and identify priorities for future research, policy, and practice.

### Strengths and Limitations

This review has several strengths. It is guided by a rigorous scoping review methodology informed by Joanna Briggs Institute guidance and supported by a comprehensive multi-database search strategy, dual independent screening, calibrated data charting, and structured analytic synthesis. It is further strengthened by an integrated theoretical framework drawing on CRT, CRL, intersectionality, and ecosocial theory to support analysis of structural racism, institutional power, differential experience, and embodiment in health contexts affecting ACB populations. In addition, the review addresses an important gap in an emerging field by synthesizing how CRL has been defined, conceptualized, and operationalized in relation to health equity for ACB populations in HICs.

Several limitations should also be acknowledged. First, the emergent and potentially fragmented nature of CRL scholarship may make it difficult to identify and consistently chart all relevant definitions, dimensions, and operationalizations across studies. Second, the inclusion of heterogeneous evidence sources with varying levels of methodological detail and reporting may pose challenges for consistent data extraction despite piloting efforts. Third, the literature specific to ACB populations may be limited, and some potentially relevant studies may address broader racialized populations without providing sufficiently disaggregated findings for inclusion. Finally, the review will be limited to sources available in English and French, which may introduce language bias and exclude potentially relevant scholarship published in other languages.

### Dissemination and Future Directions

The completed review will provide a foundation for future scholarship on CRL in health-related contexts involving ACB populations in HICs. By clarifying how CRL is currently defined, the dimensions and practices associated with it, and the ways in which it has been operationalized, the review will help inform the development of more conceptually coherent and structurally grounded research, policy, and practice. It may also help refine CRL-informed approaches in areas such as health professional education, organizational accountability, policy development, and community-based health equity work.

Future research may build on these findings by developing more explicit CRL frameworks for health contexts, examining barriers to and facilitators of operationalization, and exploring how CRL-informed approaches can be evaluated in relation to institutional change and health equity outcomes. Additional work may also be needed to strengthen indicators, measures, and practice-based applications of CRL while ensuring continued attention to anti-Black racism, intersectional inequities, and the structural conditions through which health inequities are produced and sustained.

## Data Availability

Data sharing is not applicable to this paper as no datasets were generated or analyzed during this study.

## Appendix

Multimedia Appendix 1 Initial search strategy.

## Abbreviations

ACB: African, Caribbean, and Black
CRL: critical racial literacy
CRT: critical race theory
HIC: high-income country
PRISMA-ScR: Preferred Reporting Items for Systematic Reviews and Meta-Analyses extension for Scoping Reviews
