# Supplementary material for: Integrating Critical Racial Literacy to Advance Health Equity Among African, Caribbean, and Black Populations in High-Income Countries: Protocol for a Scoping Review
Source: JMIR Res Protoc. 2026 Jun 11;15:e79361. doi: 10.2196/79361 (PMC13305473; doi:10.2196/79361)
Supplement: Multimedia Appendix 1 [file resprot_v15i1e79361_app1.doc]

**Multimedia Appendix 1. Medline OVID search strategy**

**Database:** Ovid MEDLINE ALL

**Search period:** From database inception to March 31, 2026

### **Set 1. Population: African, Caribbean, and Black populations**

1. exp Black People/
2. exp African Americans/
3. exp Emigrants and Immigrants/
4. (African* or Caribbean* or Black or Blacks or Afro* or "Afro-Caribbean*" or "African Canadian*" or "Black Canadian*" or "African Nova Scotian*" or "African diaspora" or "Caribbean diaspora" or "Black diaspora" or "African descent" or "Caribbean descent" or ACB or "sub-Saharan African*" or "Afro-Latin*").ti,ab,kf.
5. 1 or 2 or 3 or 4

### **Set 2. Phenomenon of Interest: Critical Racial Literacy and related concepts**

1. Racism/
2. ("critical racial literacy" or "critical race literac*" or "racial literacy" or CRL or "critical consciousness" or "antiracist action" or "anti-racist action" or "antiracist praxis" or "anti-racist praxis" or "structural racism" or "systemic racism" or "institutional racism" or "anti-Black racism" or "race evasiveness" or "racial evasiveness" or "racial silence" or "power analysis*" or "racial power" or "power hierarch*" or "power asymmetr*" or "structural analysis").ti,ab,kf.
3. 6 or 7

### **Set 3. Health-related context**

1. exp Health Education/
2. exp Delivery of Health Care/
3. exp Health Services/
4. exp Health Promotion/
5. exp Program Development/
6. exp Public Health/
7. exp Health Policy/
8. ("health education" or "health program*" or "health training" or "professional development" or healthcare or "health care" or "health system*" or "health service*" or "public health" or "health policy" or "health equity" or "health promotion" or "clinical care" or "health professional education" or "health professional training" or "anti-racism training" or "antiracism training" or "equity training" or "diversity training" or "inclusion training").ti,ab,kf.
9. 9 or 10 or 11 or 12 or 13 or 14 or 15 or 16

### **Set 4. High-income country context**

1. exp Developed Countries/
2. (high income countr* or HIC or HICs or "developed countr*" or "developed nation*" or "industrialized countr*" or "industrialised countr*" or OECD).ti,ab,kf.
3. (Canada or "United States" or "United Kingdom" or Australia or "New Zealand" or Ireland or France or Germany or Belgium or Netherlands or Luxembourg or Switzerland or Austria or Sweden or Norway or Denmark or Finland or Iceland or Italy or Spain or Portugal or Greece or Japan or "South Korea" or Singapore or Israel).ti,ab,kf.
4. 18 or 19 or 20

### **Final search combination**

1. 5 and 8 and 17 and 21

**Transferability and Grey Literature Adaptation**
This MEDLINE strategy (Set 1 AND Set 2 AND Set 3 AND Set 4) will be refined and translated to other peer-reviewed databases (EMBASE, CINAHL, PsycINFO, Scopus, ERIC) per the three-step approach outlined in the protocol. For grey literature searches, such as in CADTH Grey Matters, OpenGrey, and targeted HIC health organization websites, the same Boolean keyword structure applies using title/abstract/full-text fields. Searches will run from inception to March 31, 2026, with hand-searching of reference lists where relevant.
